# Determinants of bystander removal attempts in witnessed foreign body airway obstruction: a prospective nationwide multicenter observational study from the MOCHI registry

**DOI:** 10.1016/j.resplu.2026.101342

**Published:** 2026-04-27

**Authors:** Yutaka Igarashi, Tatsuya Norii, Hatsumi Nakanishi, Ryotaro Suga, Ryuta Nakae, Shoji Yokobori, MOCHI Investigators, Shoichi Yoshiike, Kosuke Shiroto, Tatsuho Kobayashi, Hiroko Kikuchi, Riko Wakisaka, Yosuke Homma, Masakazu Obayashi, Yasuo Shichinohe, Daiki Sunada, Ryu Sugimoto, Atsushi Koyama, Tomomi Iwashita, Masato Miyauchi, Tadashi Kaneko, Kazushige Inoue, Eiju Hasegawa, Nobuhiro Sato, Kiyoshi Matsuda, Jun-ichi Inoue, Takashi Tagami, Tomohiro Hattori, Toru Miike, Mayuko Koba, Kosuke Nakano, Naoki Tominaga, Eichi Narimatsu, Naofumi Bunya, Satoshi Yamanouchi, Hiroshi Takase, Tetsuya Matsuoka, Shota Nakao, Sung-Ho Kim, Toru Hifumi, Kazuhito Tamehiro, Yuji Tokuda, Mariko Sugita, Yoshihide Nakagawa, Hirotsugu Kaneshima, Taku Funakoshi, Ririko Kuwana, Kenta Ishii, Satoshi Takao, Sunao Yamauchi

**Affiliations:** 1 Aizawa Hospital; 2 Aizu Chuo Hospital; 3 Ashikaga Red Cross Hospital; 4 Chiba Kaihin Municipal Hospital; 5 Chutoen General Medical Center; 6 Hokkaido Medical Center; 7 Hyogo Prefectural Tamba Medical Center; 8 Iwaki City Medical Center; 9 Japanese Red Cross Society Nagano Hospital; 10 Kochi Medical School Hospital; 11 Mie University Hospital; 12 National Disaster Medical Center; 13 Niigata City General Hospital; 14 Nippon Medical School Musashi-Kosugi Hospital; 15 Otemachi Hospital; 16 Saga University Hospital; 17 Saitama City Hospital; 18 Sapporo Medical University Hospital; 19 Sendai City Hospital; 20 Senshu Trauma and Critical Care Center; 21 St. Luke’s International Hospital; 22 St. Mary’s Hospital; 23 Tokai University Hachioji Hospital; 24 Tokai University Hospital; 25 Tokyo Bay Urayasu Ichikawa Medical Center; 26 Toyohashi Municipal Hospital; 27 Unnan City Hospital; 28 Yuuai Medical Center; a Department of Emergency and Critical Care Medicine, Nippon Medical School, Tokyo, Japan; b Department of Emergency Medicine, University of New Mexico, Albuquerque, USA; c Department of Education and Training, St. Luke’s International Hospital, Tokyo, Japan

**Keywords:** Airway obstruction, Foreign bodies, First aid, Bystander, Registry

## Abstract

**Background:**

Foreign body airway obstruction (FBAO) is a time-critical emergency. Although bystander removal maneuvers can improve outcomes, they are not consistently attempted even when events are witnessed. We aimed to identify patient-, witness-, and setting-related factors associated with the absence of bystander removal attempts in witnessed FBAO.

**Methods:**

We analyzed a prospective, nationwide, multicenter cohort from the MOCHI registry in Japan, including patients with witnessed FBAO. The primary outcome was absence of a bystander removal attempt. Multivariable logistic regression with multiple imputation for missing data was used to examine associations with patient age, sex, eating function, location (home, care facility, public), and witness/bystander age and sex.

**Results:**

Among 325 witnessed FBAO events, 193 (59%) involved a removal attempt and 132 (41%) did not. The median patient age was 81 years. Attempt rates varied by location: 82% in care facilities, 52% at home, 43% in public places. Witnesses/bystanders were significantly older in events without an attempt (median 60 vs. 50 years). Older witness/bystander age was independently associated with higher odds of no attempt (OR 1.39 per 10-year increase; 95% CI 1.14–1.70). Compared with home, no attempt was less frequent in care facilities (OR 0.43; 95% CI 0.20–0.93) and more frequent in public places (OR 2.11; 95% CI 1.08–4.10).

**Conclusions:**

In witnessed FBAO, removal attempts were less likely when witnesses were older and when events occurred in public places. These findings may help inform age-tailored and setting-specific first-aid strategies to increase bystander intervention.

## Introduction

Foreign body airway obstruction (FBAO) is a time-critical emergency and a leading cause of accidental death, particularly among older adults.^1^ With rapid population aging worldwide, the absolute number of individuals at risk of FBAO is increasing. Age-related physiological and clinical changes—such as decline in swallowing function, reduced cough reflex, frailty, dentition problems, polypharmacy, and cognitive impairment—predispose older adults to FBAO during eating. Consistent with this growing burden, FBAO accounted for approximately 120,000 deaths worldwide in 2023.^2^

In severe FBAO, hypoxia may rapidly lead to loss of consciousness and cardiac arrest. Early recognition and immediate bystander removal attempts are therefore essential to improve survival and neurological outcomes.^3^, ^4^, ^5^ Although such bystander interventions have been independently associated with approximately two-fold higher odds of favorable neurological outcome compared with no intervention, nearly half of witnessed FBAO events receive no removal attempts.^3^ Therefore, understanding when and where bystander removal attempts are not performed has direct public-health relevance.

In Japan, basic life support education is widely disseminated through school- and community-based programs and national resuscitation guidance; however, recognition of severe FBAO and initiation of foreign-body removal maneuvers may be less embedded in lay practice than cardiopulmonary resuscitation and automated external defibrillator actions. Accordingly, identifying determinants of no bystander removal attempt may inform targeted public-health strategies. Using a nationwide, prospective multicenter registry in Japan, we aimed to identify patient-, witness-, and setting-related factors associated with the absence of bystander foreign-body removal attempts among witnessed FBAO events.

## Methods

### Study design and setting

This was a prospective, nationwide, multicenter observational study in Japan using the Multicenter Observational CHoking Investigation (MOCHI) registry, for which data collection was conducted according to previously published and registered study protocol.^6^ We included patients with FBAO transported to the participating hospitals from April 2020 to March 2023. For this analysis, patients were excluded if the incident was not witnessed or unknown because witness status fundamentally determines the opportunity for early recognition and on-scene bystander action. This approach is consistent with resuscitation registry frameworks that treat witness status as a core determinant of bystander response and outcomes.^7^ In Japan, when a choking event is reported to the emergency dispatch center (119 call), dispatchers routinely provide standardized telephone instructions on foreign-body removal techniques, including back blows and abdominal thrusts, to callers before emergency medical services (EMS) arrival. The study protocol was approved by the Institutional Review Board of Nippon Medical School Hospital (Approval No. B-2019-019) and the ethics committees of all participating hospitals. This manuscript follows the Strengthening the Reporting of Observational Studies in Epidemiology guideline for observational studies.^8^

### Data collection and variables

Data were collected in accordance with standardized MOCHI protocol.^6^ Treating physicians in the emergency department obtained information on the circumstances of the FBAO event using a structured interview form at each participating site, as specified in the MOCHI protocol. Information was obtained from witnesses and/or bystanders when available and supplemented by EMS personnel and official EMS records. Interviews were conducted as early as feasible after hospital arrival and were primarily in person; when witnesses were not present, information could be obtained by telephone.

Independent variables included patient age (per 10-year increment), patient sex, eating function (independent vs. not independent), accident location (home, care facility, or public), and age and sex of the recorded witness (per 10-year increment). Eating function was defined as baseline feeding independence rather than a direct measure of swallowing function. “Witness” referred to the recorded person who observed the event when no bystander removal attempt was performed, and to the person who performed the intervention when a bystander removal attempt was made. A “bystander removal attempt” was defined as any recorded attempt by the bystander to relieve the obstruction using one or more of the following maneuvers on the scene regardless of success or failure: back blows, abdominal thrusts, chest thrusts, finger sweep/manual removal, suction, or other documented removal actions. Attempts by staff in care facilities were considered bystander attempts in this study because they represent on-scene responders before definitive in-hospital care. This definition was intended to capture on-scene response broadly across settings rather than lay bystanders alone. To mitigate potential multicollinearity between accident location and witness characteristics, we categorized the location into three groups: home, care facility, and public places, a pragmatic classification for targeted prevention. This classification was adopted because incidents at home were predominantly witnessed by family members, whereas those at care facilities were witnessed by healthcare staff (Table S1).

### Outcome

The primary outcome was the absence of a bystander removal attempt, defined operationally as the absence of any recorded on-scene removal action by a bystander rather than a direct measure of recognition, willingness, or ability to intervene.

### Statistical analysis

Continuous variables were presented as medians with interquartile ranges (IQR), and categorical variables were expressed as numbers and percentages. We compared baseline characteristics between groups using the Mann–Whitney *U* test for continuous variables and Pearson’s chi-squared test or Fisher’s exact test for categorical variables.

We employed a multivariable logistic regression model to identify factors associated with the absence of bystander removal attempts. We selected these variables a priori based on clinical relevance and feasibility for targeted public-health interventions. Some variables, such as food type, were not included in the multivariable model because they were descriptive and location-specific in nature, closely correlated with event location, and had sparse categories. To account for within-hospital correlation, we calculated hospital-level cluster-robust standard errors. To address missing data, we performed multiple imputation by chained equations generating 20 imputed datasets. The imputation model included all covariates in the analysis model and the outcome variable. Because missingness was limited to witness sex and the remaining covariates were complete, we considered the missing-at-random assumption to be plausible, although this assumption cannot be verified empirically. Each imputed dataset was analyzed using logistic regression, and estimates were pooled using Rubin’s rules.

We conducted prespecified sensitivity analyses using (1) complete-case analyses and (2) exclusion of care-facility events to evaluate the robustness of our findings. Model diagnostics included an assessment of multicollinearity using the variance inflation factor (VIF). All statistical analyses were performed using R 4.3.1 (The R Foundation for Statistical Computing, Vienna, Austria).

## Results

**Fig. 1:**
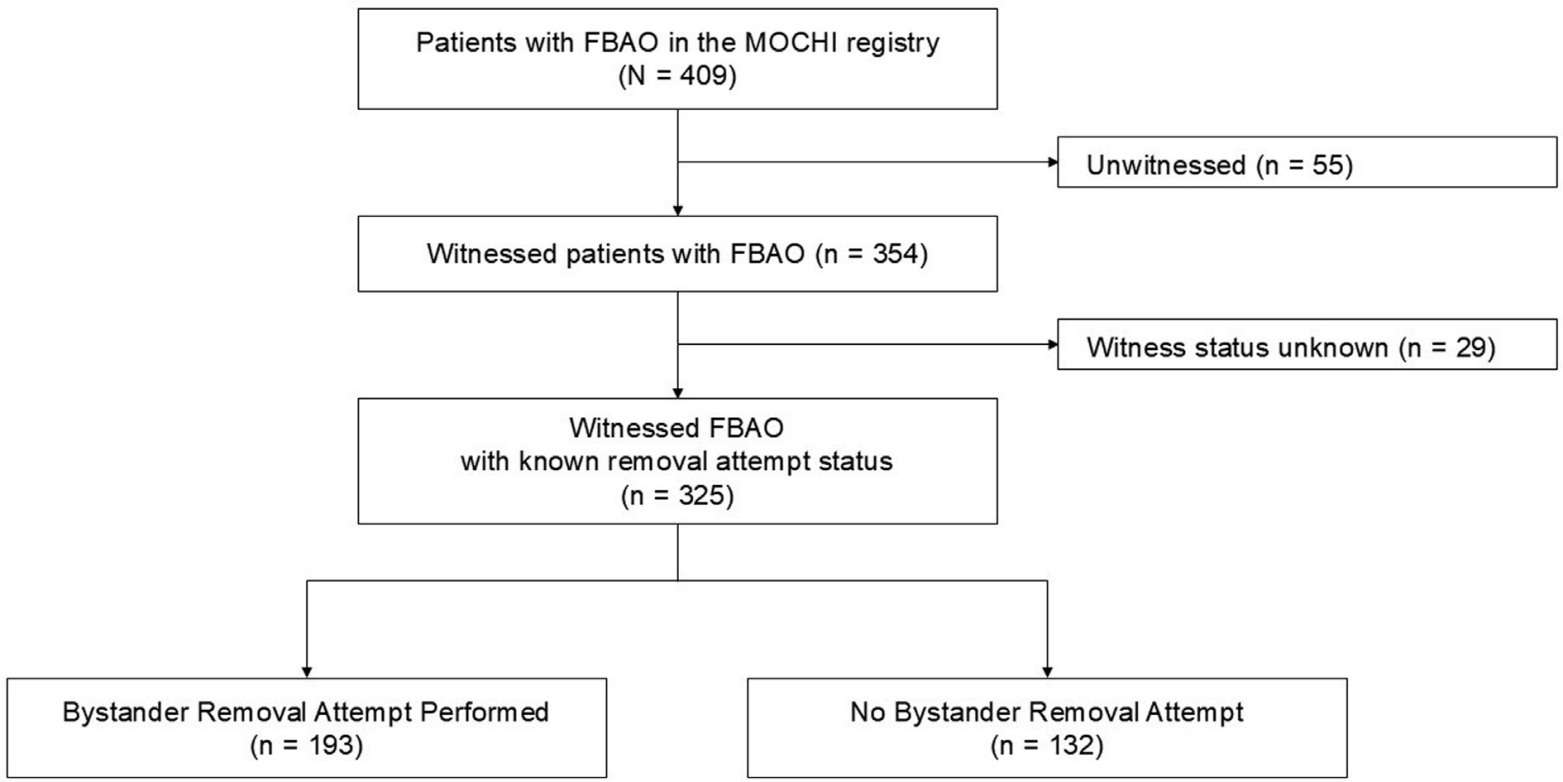
**Flow diagram of study population selection**. FBAO, foreign body airway obstruction; MOCHI, Multicenter Observational CHoking Investigation.

A total of 325 patients with witnessed FBAO were included in the analysis (Fig. 1). Of these, a bystander removal attempt was performed in 193 cases (59%) and was absent in 132 cases (41%). The baseline characteristics of the patients and witnesses are summarized in Table 1. The median patient age was 81 years (IQR: 73–87) in both groups. Removal attempts were more frequent in care facilities (82%) than at home (52%) or in public places (43%). The median age of the recorded witness/bystander was significantly higher in the group where removal attempts were absent (60 years; IQR: 50–75) compared to the group where attempts were performed (50 years; IQR: 40–60) (Table 1). Witness/bystander sex was missing in 62 cases (19%) and was imputed; other covariates were complete.

**Table 1:** Baseline characteristics of witnessed FBAO events by presence of bystander removal attempt.

Variables	**Overall**	**Removal attempt** **(*n* = 193)**	**No removal attempt** **(*n* = 132)**	***P* value**
Age, median (IQR)	81 (73, 87)	81 (73, 87)	81 (72, 86)	0.8
Sex				0.089
Male	181 (56%)	100 (52%)	81 (61%)	
Female	144 (44%)	93 (48%)	51 (39%)	
Eating function				0.089
Independent	245 (75%)	139 (72%)	106 (80%)	
Not independent	80 (25%)	54 (28%)	26 (20%)	
Location				<0.001
Home	184	95 (52%)	89 (48%)	
Care facility	95	78 (82%)	17 (18%)	
Public	46	20 (43%)	26 (57%)	
Witness/bystander age, median (IQR)	50 (40, 70)	50 (40, 60)	60 (50, 75)	<0.001
Witness/bystander sex				0.7
Male	95 (36%)	52 (35%)	43 (37%)	
Female	168 (64%)	96 (65%)	72 (63%)	

IQR, interquartile range. Witness sex was missing in 62 cases (19%); percentages for witness sex are based on non-missing data.

**Fig. 2:**
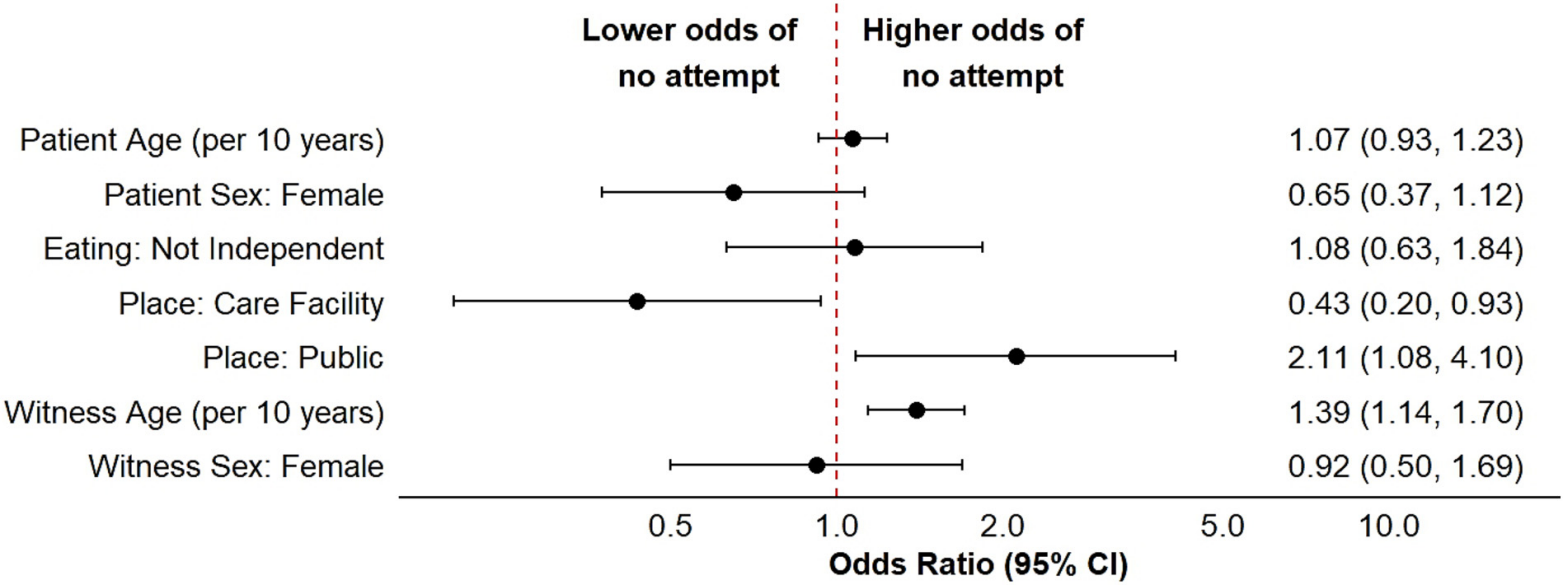
**Factors associated with absence of bystander removal attempts in witnessed FBAO (multivariable analysis)**. Forest plot of adjusted odds ratios (ORs) with 95% confidence intervals from a multivariable logistic regression model for the primary outcome (no bystander removal attempt). FBAO, foreign body airway obstruction; OR, odds ratio; CI, confidence interval.

In the multiple imputation analysis, using logistic regression with hospital-level cluster-robust standard errors, higher witness age (per 10-year increase) was significantly associated with the absence of removal attempts (OR 1.39; 95% CI 1.14–1.70). Compared to incidents at home, the absence of attempts was significantly less frequent in care facilities (OR 0.43; 95% CI 0.20–0.93) and more frequent in public places (OR 2.11; 95% CI 1.08–4.10) (Fig. 2). Sensitivity analysis using complete-case data and excluding care-facility events yielded materially similar odds ratios, supporting the robustness of the main findings (Table S2). Multicollinearity was assessed using the generalized variance inflation factor (GVIF). All predictors showed low GVIF values (<1.2), suggesting negligible multicollinearity (Table S3).

Specific characteristics of the FBAO incidents varied by location (Table 2). Food types differed across settings (mochi at home, rice in care facilities, and meat in public places), and alcohol consumption was more frequent in public places (20%). Regarding initial bystander maneuvers, back blows were common across all locations, whereas suction was more frequently used in care facilities (38%) and abdominal thrusts were more common in care facilities (12%) and public places (13%) than at home (4%). In care-facility events, 97% of witnesses were healthcare or care staff, and 82% of events involved a bystander removal attempt (Table S1). In an exploratory subgroup analysis of public-location events, 21 of 31 restaurant events (68%) had no recorded removal attempt (Table S4).

**Table 2:** Location-specific characteristics of witnessed FBAO events: suspected foreign body and initial bystander maneuvers.

**Variables**	**Home184** **(57%)**	**Care facility95** **(29%)**	**Public46** **(14%)**
**Food**			
Rice	30 (16%)	30 (32%)	6 (13%)
Rice cake (mochi)	44 (24%)	1 (1%)	3 (7%)
Bread	18 (10%)	9 (10%)	0 (0%)
Meat	23 (13%)	15 (16%)	17 (37%)
Fish	7 (4%)	4 (4%)	2 (4%)
Sushi	6 (3%)	2 (2%)	2 (4%)
Jelly	3 (2%)	4 (4%)	1 (2%)
Vegetable	15 (8%)	9 (11%)	2 (4%)
Fruit	8 (4%)	6 (6%)	1 (2%)
Potato	2 (1%)	0 (0%)	1 (2%)
Noodle	8 (4%)	1 (1%)	3 (7%)
Alcohol consumption	8 (4%)	0 (0%)	9 (20%)
**Initial maneuver**			
Abdominal thrusts	7 (4%)	11 (12%)	6 (13%)
Back blows	52 (28%)	23 (24%)	8 (17%)
Chest thrusts	14 (8%)	4 (4%)	3 (7%)
Finger sweep	26 (14%)	12 (13%)	6 (13%)
Suction	34 (18%)	36 (38%)	11 (24%)
Vacuum cleaner	1 (1%)	0 (0%)	0 (0%)

FBAO, foreign body airway obstruction.

## Discussion

In this nationwide prospective cohort of witnessed FBAO events, increasing older witness age was independently associated with the absence of bystander foreign-body removal attempts. Event location was also an independent key determinant: compared with events occurring at home, attempts were more frequent in care facilities and less frequent in public places. Collectively, these findings identify settings in which bystander FBAO first aid is least likely to be initiated, thereby highlighting actionable targets for training and public-health interventions.

The high frequency of removal attempts in care facilities should be interpreted in light of witness composition and institutional context. In our cohort, most care-facility events were witnessed by healthcare or care staff, suggesting that the observed association may have reflected responder training, role expectations, and local workflows rather than the setting itself. Accordingly, comparisons between home and care-facility settings should not be interpreted as a simple contrast between locations alone.

A clinically important contrast emerges, however, when our finding regarding public-location events is considered alongside the out-of-hospital cardiac arrest (OHCA) literature. Multiple large studies have reported better outcomes for OHCA in public locations, partly attributable to higher rates of bystander CPR and public-access defibrillation.^9^, ^10^, ^11^, ^12^ In the present study, however, public places were associated with a lower likelihood of bystander removal attempts for FBAO. Consistent with this, an exploratory analysis showed that 21 of 31 restaurant events (68%) involved no recorded removal attempt, suggesting that certain public settings may warrant further investigation. This association may reflect not only the public setting itself but also witness unfamiliarity, ambiguity, or delayed recognition of severe FBAO. One possible explanatory framework is the bystander-effect literature; however, these mechanisms were not directly measured in our study, and the following interpretations should be regarded as hypothesis-generating. Public settings may promote diffusion of responsibility, uncertainty about who should act, or fear of negative evaluation.^13^, ^14^ In OHCA, these effects may be attenuated because the expected response consists of highly standardized and widely disseminated actions (e.g., call for help, start compressions, use an AED)^4^, ^5^ and because visible enabling infrastructure exists, including AED placement and signage.^10^, ^11^ Moreover, the decision threshold for initiating CPR is comparatively clear (unresponsiveness with abnormal or absent breathing), and CPR has become a socially normative lifesaving behavior.

FBAO may also be more difficult for laypersons to recognize rapidly. Severe FBAO can present with ineffective cough, inability to speak, cyanosis, altered mental status, or apnea—features that overlap with other acute conditions and may complicate rapid recognition.^15^ Abrupt loss of voice or effective cough during or immediately after eating (“silent choking”) may represent one possible explanation for delayed recognition in some cases, although this was not directly assessed in our study.^16^ In addition, some maneuvers—especially abdominal thrusts—could be perceived as invasive and require close physical contact, which might increase hesitation in public and amplify bystander-effect dynamics. These considerations are hypothesis-generating and suggest that improving bystander responses to FBAO in public settings may benefit from strategies analogous to those used for OHCA. Potential approaches, which warrant future evaluation, include simplified recognition cues for severe FBAO, standardized messaging, venue-specific staff response protocols, caregiver-focused teaching, and repeated skills refreshers for older adults.

The association between older witness/bystander age and no removal attempt may reflect a combination of fewer training opportunities, physical limitations, and lower self-efficacy—factors that have also been described for bystander CPR among older adults.^17^ Older witnesses may anticipate difficulty performing maneuvers correctly, which could inhibit initiation.^18^ However, these factors should be interpreted as possible explanations rather than direct findings. Accordingly, witness age may function as a composite marker of training exposure, physical capacity, and confidence rather than a purely demographic characteristic.

We also observed location-specific patterns in the initial maneuvers. Suction was frequently used in care facilities, consistent with equipment availability and routine clinical practice.^19^ However, guideline-recommended first aid for severe FBAO emphasizes back blows and abdominal thrusts. Over-reliance on suction may delay recommended maneuvers when severe FBAO is suspected. Training in care settings should therefore reinforce early implementation of recommended maneuvers while clarifying the appropriate role and limitations of suction.

These data suggest substantial opportunities to improve bystander response to FBAO in public places and among older witnesses. In our cohort, approximately two-thirds of restaurant events involved no removal attempt, suggesting that restaurant-focused staff training may be a reasonable strategy to evaluate in future studies. At home, nearly half of events involved no attempt, indicating that repeated training for older adults and family caregivers may also be beneficial.

This study has several limitations. First, as an observational study, the findings demonstrate associations and do not establish causality. Second, the cohort was restricted to FBAO events presenting to the ED of participating hospitals; thus, FBAO cases successfully resolved on scene by bystander maneuvers were not captured and may be underrepresented, potentially affecting sample representativeness and generalizability. Third, interview timing/mode were not mandatory fields; therefore, classification of removal attempts and maneuvers relied on recorded information and may be subject to misclassification or incomplete detail. Fourth, we could not reliably distinguish complete from partial obstruction; although the primary outcome was the presence of any recorded removal attempt, variability in obstruction severity may have influenced symptom recognition and propensity to initiate first aid. Therefore, “no bystander removal attempt” should not be interpreted solely as a behavioral failure, because in some cases it may also have reflected diagnostic uncertainty, perceived infeasibility, or clinical situations in which intervention was not clearly indicated or recognized. This may have introduced outcome misclassification. In addition, residual confounding from unmeasured factors—prior first-aid training, the relationship between the witness and patient, the number of bystanders present, and the initial clinical presentation—cannot be excluded and may have influenced both recognition of severe FBAO and the likelihood of intervention. Finally, the study period overlapped with the COVID-19 pandemic, which may have influenced willingness to perform close-contact maneuvers and local workflows, potentially affecting observed intervention patterns.^20^

## Conclusion

In a nationwide prospective registry of witnessed FBAO events, bystander removal attempts were less likely in public places and when witnesses were older. These findings highlight contextual and witness-related differences in bystander response to witnessed FBAO and may help inform future studies and public-health strategies aimed at increasing removal attempts, which may contribute to improved outcomes.

## CRediT authorship contribution statement

**Yutaka Igarashi:** Writing – original draft, Project administration, Methodology, Investigation, Funding acquisition, Formal analysis, Data curation, Conceptualization. **Tatsuya Norii:** Writing – review & editing, Supervision, Project administration, Methodology, Investigation, Funding acquisition. **Hatsumi Nakanishi:** Formal analysis, Data curation. **Ryotaro Suga:** Visualization, Investigation. **Ryuta Nakae:** Writing – review & editing, Conceptualization. **Shoji Yokobori:** Writing – review & editing, Supervision, Funding acquisition.

## Funding

This work was supported by the Ministry of Education, Culture, Sports, Science and Technology (MEXT), Japan, through the Program for Developing Advanced Medical Human Resources; the Japanese Association for Acute Medicine; Taiju Life Social Welfare Foundation.

## Declaration of competing interest

The authors declare that they have no known competing financial interests or personal relationships that could have appeared to influence the work reported in this paper.

## Appendix A MOCHI Investigators (in alphabetical order by institution)

Aizawa Hospital: Shoichi Yoshiike, MD; Kosuke Shiroto, MD.

Aizu Chuo Hospital: Tatsuho Kobayashi, MD.

Ashikaga Red Cross Hospital: Hiroko Kikuchi, MD; Riko Wakisaka, MD.

Chiba Kaihin Municipal Hospital: Yosuke Homma, MD.

Chutoen General Medical Center: Masakazu Obayashi, MD.

Hokkaido Medical Center: Yasuo Shichinohe, MD; Daiki Sunada, MD.

Hyogo Prefectural Tamba Medical Center: Ryu Sugimoto, MD.

Iwaki City Medical Center: Atsushi Koyama, MD.

Japanese Red Cross Society Nagano Hospital: Tomomi Iwashita, MD.

Kochi Medical School Hospital: Masato Miyauchi, MD.

Mie University Hospital: Tadashi Kaneko, MD.

National Disaster Medical Center: Kazushige Inoue, MD; Eiju Hasegawa, MD.

Niigata City General Hospital: Nobuhiro Sato, MD.

Nippon Medical School Musashi-Kosugi Hospital: Kiyoshi Matsuda, MD; Jun-ichi Inoue, MD; Takashi Tagami, MD.

Otemachi Hospital: Tomohiro Hattori, MD.

Saga University Hospital: Toru Miike, MD; Mayuko Koba, MD.

Saitama City Hospital: Kosuke Nakano, MD; Naoki Tominaga, MD.

Sapporo Medical University Hospital: Eichi Narimatsu, MD; Naofumi Bunya, MD.

Sendai City Hospital: Satoshi Yamanouchi, MD; Hiroshi Takase, MD.

Senshu Trauma and Critical Care Center: Tetsuya Matsuoka, MD; Shota Nakao, MD; Sung-Ho Kim, MD.

St. Luke’s International Hospital: Toru Hifumi, MD.

St. Mary’s Hospital: Kazuhito Tamehiro, MD; Yuji Tokuda, MD.

Tokai University Hachioji Hospital: Mariko Sugita, MD.

Tokai University Hospital: Yoshihide Nakagawa, MD; Hirotsugu Kaneshima, MD.

Tokyo Bay Urayasu Ichikawa Medical Center: Taku Funakoshi, MD; Ririko Kuwana, MD.

Toyohashi Municipal Hospital: Kenta Ishii, MD.

Unnan City Hospital: Satoshi Takao, MD.

Yuuai Medical Center: Sunao Yamauchi, MD.

## Appendix B Supplementary material

Supplementary Tables Supplementary material. Additional tables supporting the main analyses. Location-specific witness characteristics, sensitivity analyses, multicollinearity diagnostics, and exploratory public-location subgroup analyses.
