# Supplementary material for: Determinants of bystander removal attempts in witnessed foreign body airway obstruction: a prospective nationwide multicenter observational study from the MOCHI registry
Source: Resusc Plus. 2026 Apr 27;29:101342. doi: 10.1016/j.resplu.2026.101342 (PMC13158428; doi:10.1016/j.resplu.2026.101342)
Supplement: Supplementary Tables — Supplementary material. Additional tables supporting the main analyses. Location-specific witness characteristics, sensitivity analyses, multicollinearity diagnostics, and exploratory public-location subgroup analyses. [file mmc1.docx]

**Supplementary Table**

**Table S1**

**Witness relationship to the patient by event location in witnessed FBAO**

|  | Location | | | Total  (n= 325) |
| --- | --- | --- | --- | --- |
|  | Home  (n = 184) | Care facility  (n = 95) | Public  (n = 46) |  |
| Witness relationship |  |  |  |  |
| Family | 178 (97%) | 0 (0%) | 20 (43%) | 198 (61%) |
| Friend | 0 (0%) | 1 (1%) | 5 (11%) | 6 (2%) |
| Healthcare workers | 4 (2%) | 92 (97%) | 12 (26%) | 108 (33%) |
| Other | 2 (1%) | 2 (2%) | 9 (20%) | 13 (4%) |

FBAO, foreign body airway obstruction.

**Table S2**

**Sensitivity analyses for factors associated with absence of bystander removal attempts in witnessed FBAO**

|  | Sensitivity Analysis | | Multiple Imputation Analysis | |
| --- | --- | --- | --- | --- |
|  | Complete-Case Analysis | Excluding Care Facility | |  |
| Patient age (per 10 years) | 1.06 (0.89, 1.28) | 1.08 (0.90, 1.30) | | 1.02 (0.72, 1.46) |
| Sex |  |  | |  |
| Male | Ref. | Ref. | | Ref. |
| Female | 0.71 (0.39, 1.27) | 0.66 (0.37, 1.19) | | 1.05 (0.95, 1.17) |
| Eating function |  |  | |  |
| Independent | Ref. | Ref. | | Ref. |
| Not independent | 1.11 (0.55, 2.24) | 1.11 (0.62, 2.00) | | 0.65 (0.36, 1.19) |
| Location |  |  | |  |
| Home | Ref. | Ref. | | Ref. |
| Care facility | 0.44 (0.17, 1.09) | N/A | | 0.42 (0.25, 0.71) |
| Public | 2.28 (1.06, 5.06) | 1.93 (1.05, 3.57) | | 2.15 (1.22, 3.80) |
| Witness age (per 10 years) | 1.49 (1.23, 1.84) | 1.36 (1.08, 1.71) | | 1.40 (1.18, 1.66) |
| Witness sex |  |  | |  |
| Male | Ref. | Ref. | | Ref. |
| Female | 0.91 (0.50, 1.64) | 0.95 (0.49, 1.84) | | 0.93 (0.50, 1.75) |

N/A, not applicable; Ref, reference.

**Table S3**

**Multicollinearity assessment using the generalized variance inflation factor**

| **Predictor** | **GVIF** | **Df** | **Adjusted GVIF** |
| --- | --- | --- | --- |
| Patient age (per 10 years) | 1.234 | 1 | 1.111 |
| Patient sex | 1.191 | 1 | 1.091 |
| Eating function | 1.090 | 1 | 1.044 |
| Event location group (home/care facility/public)* | 1.319 | 2 | 1.072 |
| Witness age (per 10 years) | 1.407 | 1 | 1.186 |
| Witness sex | 1.159 | 1 | 1.077 |

GVIF, generalized variance inflation factor. For predictors with more than one degree of freedom (Df), we report the adjusted GVIF as $\mathrm{GVIF}^{1/(2\times\mathrm{Df})}$. All adjusted GVIF values were < 1.2, suggesting negligible multicollinearity.

**Table S4**

**Witness relationship and bystander removal attempts in public-place FBAO events by specific setting**

|  | Restaurant (n= 31) | Park (n = 2) | School (n = 2) | Street (n = 1) | Other (n =10) |
| --- | --- | --- | --- | --- | --- |
| Witness attempt | 10 | 0 | 1 | 1 | 8 |
| Family | 7 | 0 | 0 | 0 | 0 |
| Friend | 0 | 0 | 0 | 0 | 1 |
| Healthcare workers | 2 | 0 | 1 | 1 | 7 |
| Others | 1 | 0 | 0 | 0 | 0 |
| No witness attempt | 21 | 2 | 1 | 0 | 2 |
